# Gut microbiota-bone marrow axis: how intestinal signals remodel hematopoiesis and immune status

**DOI:** 10.3389/fimmu.2026.1987932

**Published:** 2026-10-22

**Authors:** Xuemin Chen, Xuemei Li, Yulong Song, Xuan Liu, Jun Li

**Affiliations:** 1 Department of Gastroenterology, The First Affiliated Hospital of Chengdu Medical College, Chengdu, Sichuan, China; 2 Chengdu Medical College, Chengdu, Sichuan, China

**Keywords:** bone marrow axis, bone metabolism, gut microbiota, hematopoietic stem cells, immune regulation, systemic inflammation, toll-like receptors

## Abstract

The gut microbiota, as a complex ecological system within the host, plays a critical role not only in regulating local intestinal immunity and metabolism but also in influencing hematopoietic stem cell (HSC) function and immune cell lineage differentiation through the gut-bone marrow axis. This review comprehensively examines how the gut microbiota remodels hematopoiesis and immune status via multiple mechanisms, particularly by modulating systemic inflammation, the bone marrow microenvironment, and HSC fate decisions. Special emphasis is placed on the role of Toll-like receptor (TLR) signaling pathways in linking the gut microbiota to bone metabolism and immune regulation. The review also integrates recent insights from single-cell sequencing technologies that reveal hematopoietic lineage biases in the bone marrow and explores the development and immune memory of gut-associated B cell populations during early life. By systematically synthesizing recent advances in the interaction between gut microbiota and bone marrow hematopoiesis, this article aims to provide a theoretical foundation for understanding how gut microbiota regulate systemic immune homeostasis and bone metabolism and to identify potential therapeutic targets for future interventions.

## 1 Introduction

The human gastrointestinal tract harbors a complex and dynamic community of microorganisms collectively termed the gut microbiota, which exists in a symbiotic relationship with the host. This microbial ecosystem, dominated predominantly by bacteria, plays a fundamental role in shaping host immunity and metabolism. The gut microbiota mediates interactions with the gut epithelium and immune cells through the synthesis of vitamins and a variety of metabolic products, including short-chain fatty acids (SCFAs), bile acids, and other bioactive compounds. These microbial metabolites are instrumental in the growth and development of both innate and adaptive immune components, thereby maintaining immune homeostasis and host-microbe symbiosis (1, 2). The balance within this ecosystem, termed eubiosis, supports metabolic and immune functions, while disruptions, or dysbiosis, can lead to impaired immune responses and contribute to the pathogenesis of various diseases (1, 3). Moreover, the gut microbiota influences systemic physiological processes beyond the intestine, including neurological function via the gut-brain axis and pulmonary immune regulation through the gut-lung axis, highlighting its extensive impact on host health (4–6). The host’s diet, genetic background, and environmental exposures dynamically shape the composition and function of the gut microbiota, which in turn modulates host metabolic and immune pathways through intricate signaling networks such as Toll-like receptor (TLR) pathways and NF-κB-mediated transcriptional regulation (7–9). This bidirectional communication underscores the gut microbiota as a pivotal regulator of systemic immunity and metabolism.

Which taxa generate which metabolite, and what those metabolites act upon, is now reasonably well defined. Butyrate is produced mainly by Firmicutes of the families Lachnospiraceae and Ruminococcaceae, including *Faecalibacterium prausnitzii*, *Eubacterium rectale*, *Roseburia intestinalis* and *Anaerostipes* species, which ferment dietary fiber and also cross-feed on lactate and acetate; propionate arises largely from *Bacteroides* species, *Akkermansia muciniphila* and *Veillonella* species through the succinate and propanediol routes, whereas acetate is generated broadly, including by *Bifidobacterium* species (2, 10). Lactic acid bacteria such as *Lactobacillus* species release lactate, and tryptophan-metabolizing commensals produce indole derivatives such as indole-3-propionic acid (11). These molecules are read by a small set of receptors and enzymes: the free fatty acid receptors FFAR2 (GPR43) and FFAR3 (GPR41), the niacin receptor HCAR2 (GPR109A), the lactate receptor GPR81, the aryl hydrocarbon receptor for indole derivatives, and inhibition of histone deacetylases, which couples microbial fermentation to the epigenetic state of host cells (12). The cell types that matter for the present review lie on both sides of the intestinal–marrow interface. Butyrate and propionate drive colonic regulatory T cell generation through GPR43 and histone deacetylase inhibition (13); butyrate restrains granulopoiesis and blunts the early antimicrobial activity of neutrophils (14); GPR41 on dendritic cells limits their maturation, and its loss aggravates autoimmune pathology (15); and microbiota-derived lactate acts on GPR81 expressed by leptin receptor-positive bone marrow stromal cells to induce stem cell factor (SCF), supporting hematopoiesis and erythropoiesis (16). Depletion of the microbiota lowers these signals and reduces steady-state hematopoietic output (17, 18), indicating that the marrow reads the intestinal metabolite pool continuously rather than only under stress.

The bone marrow serves as the primary hematopoietic organ in adults, housing hematopoietic stem cells (HSCs) responsible for the lifelong generation of all blood and immune cell lineages. The maintenance of HSCs and their progenitors is intricately regulated by the specialized bone marrow microenvironment, or niche, which comprises diverse cellular constituents including mesenchymal stem cells, endothelial cells, osteoblasts, adipocytes, macrophages, and extracellular matrix components (19–21). This niche provides essential physical, chemical, and cellular cues that govern HSC quiescence, self-renewal, differentiation, and mobilization, thereby ensuring hematopoietic homeostasis (22, 23). Emerging evidence has elucidated the role of biophysical factors such as matrix stiffness and pore size in modulating HSC fate decisions, highlighting the multifaceted regulation of hematopoiesis by the bone marrow microenvironment (22). Furthermore, immune cells residing within the bone marrow, including neutrophils, macrophages, and T cells, actively participate in the regulation of hematopoiesis through direct interactions with HSCs and niche cells, as well as via secretion of cytokines and growth factors (24). Bone marrow adipocytes, once considered passive fillers, are now recognized as dynamic regulators that influence hematopoietic function positively or negatively, depending on physiological and pathological contexts such as aging, obesity, and cardiovascular disease (23, 25, 26). Additionally, systemic factors including sympathetic nervous signals and metabolic cues further modulate hematopoietic dynamics within the bone marrow niche (26, 27). Collectively, these findings underscore the complexity of the bone marrow as a highly regulated organ essential for immune competence and hematopoietic integrity.

Recent advances have begun to unravel the mechanistic links between the gut microbiota and the bone marrow hematopoietic system, conceptualized as the gut microbiota-bone marrow axis. This axis represents a critical communication pathway whereby gut-derived signals modulate hematopoiesis and immune cell development in the bone marrow, thereby influencing systemic immunity and disease susceptibility. Microbial metabolites such as SCFAs have been shown to impact neutrophil maturation and function, modulating inflammatory responses and antimicrobial activity (14). Additionally, gut microbiota composition and its metabolic outputs can regulate bone marrow hematopoiesis through immune and metabolic signaling pathways, affecting the balance of myeloid and lymphoid lineages (28, 29). Dysbiosis of the gut microbiota can disturb this axis, leading to aberrant hematopoietic responses, systemic inflammation, and increased risk of immune-related and metabolic diseases (20, 30). Nutritional interventions targeting the gut microbiota, including prebiotics, probiotics, and microbiota-accessible nutrients such as borates, have demonstrated potential in restoring healthy microbiota composition and enhancing hematopoietic and immune functions (31, 32). Moreover, the gut microbiota influences bone marrow niche components, including macrophages and mesenchymal stem cells, thereby modulating the hematopoietic microenvironment and facilitating recovery after hematopoietic stress or injury (20, 33). Understanding the molecular and cellular mechanisms underlying the gut microbiota-bone marrow crosstalk promises to unveil novel therapeutic strategies for treating immune dysregulation, hematopoietic disorders, and bone metabolism diseases.

Several recent reviews have covered parts of this ground. Fernandez Sanchez and colleagues surveyed how the intestinal microbiome shapes hematopoiesis and the management of hematological disease (34); Li et al. and Zheng et al. examined microbial regulation of benign and malignant hematopoiesis and its ecological logic (35, 36); Hirao considered microbiota effects on hematopoietic homeostasis and leukemogenesis (37); Wells et al. focused on how ageing alters the gut–bone marrow axis (38); and Indrio and Salatto reviewed the gut–bone axis from a nutritional standpoint (39). These accounts are organized either around hematopoiesis or around bone, and they largely treat the two as separate literatures. The present review differs in three respects. First, it places TLR signaling, and TLR9 in particular, at the center of the account, as the node at which microbial recognition, myeloid lineage bias and osteoclastogenesis converge, so that the hematological and skeletal consequences of dysbiosis are read as two outputs of one pathway rather than as parallel phenomena. Second, it uses single-cell transcriptomic data as mechanistic evidence for lineage remodeling rather than as descriptive illustration, and states what those datasets can and cannot yet establish. Third, it incorporates the early-life dimension — the neonatal window in which B-1a cells and gut IgA plasma cells clonally related to bone marrow plasma cells are generated — which reviews centered on adult hematopoiesis generally omit, and which is where microbial imprinting of the humoral compartment is decided. The aim is therefore not to list microbiota–hematopoiesis associations again, but to assemble them into one mechanistic account spanning development, steady state and disease.

The literature underpinning this review was assembled from PubMed and Web of Science searches combining terms for the intestinal microbiota and its metabolites (gut microbiota, microbiome, short-chain fatty acid, butyrate, lipopolysaccharide, bile acid, tryptophan or indole, dysbiosis) with terms for the marrow and immune compartments (bone marrow, hematopoietic stem cell, hematopoiesis, myelopoiesis, niche, IgA plasma cell, osteoclast, Toll-like receptor), limited to English-language reports published up to December 2025. Titles and abstracts were screened for evidence of signaling between the intestine and the bone marrow; reports confined to intestinal immunity without a hematopoietic, marrow-stromal or skeletal readout were not retained, while animal, human and *in vitro* studies were all eligible. 105 publications were included and assigned to the four themes that structure the sections below: direct metabolite- and receptor-mediated regulation of HSCs (13 references); indirect regulation through the marrow microenvironment, including stromal, inflammatory and neuro-endocrine routes (28); early-life B cell development and immune memory (13); and clinical significance and therapeutic strategies (12). The remaining 39 references provide background on microbiota and marrow biology and are cited in this Introduction. A study cited under more than one theme is counted once, at the section in which it is first discussed.

In summary, the gut microbiota and bone marrow represent two interdependent systems whose interactions critically shape hematopoietic and immune homeostasis. The gut microbiota serves as a dynamic regulator of host immunity and metabolism, while the bone marrow provides the essential niche for hematopoietic stem cell maintenance and immune cell generation. The emerging concept of the gut microbiota-bone marrow axis highlights the significance of gut-derived signals in remodeling bone marrow hematopoiesis and immune states. Deciphering the complex signaling pathways and cellular interactions involved in this axis is essential for developing innovative interventions targeting immune-related diseases, hematological disorders, and metabolic bone diseases. Continued research integrating microbiology, immunology, hematology, and metabolism will advance our understanding of this axis and pave the way for precision medicine approaches to modulate host-microbiota symbiosis and hematopoietic function for improved health outcomes.

## 2 Gut microbiota–bone marrow axis: mechanisms, development and clinical translation

### 2.1 Direct regulation of bone marrow hematopoietic stem cell function by the gut microbiota

#### 2.1.1 Interaction between microbial metabolites and hematopoietic stem cells

The gut microbiota produces a variety of metabolites, notably SCFAs and lipopolysaccharide (LPS), which enter the systemic circulation and act on the bone marrow microenvironment, thereby modulating HSC function. SCFAs — acetate, propionate and butyrate — are fermentation products of dietary fiber and act as signaling molecules that regulate cytokine production and epigenetic modification within HSCs, influencing lineage commitment and hematopoietic homeostasis (12). Butyrate contributes to the expansion of regulatory T cells through GPR43 engagement and histone deacetylase inhibition (13), and restrains granulopoiesis while blunting early neutrophil antimicrobial activity in the lung (14), so that its net effect on hematopoiesis is both direct and immune-mediated. Yan et al. showed that this dependence extends to the steady state: microbiota-derived metabolites, among them phosphatidylcholine and γ-glutamylalanine, sustain the tonic type I interferon signaling required for normal hematopoiesis, and antibiotic depletion of the microbiota suppresses it (18). In the same vein, Zhang et al. found that intestinal signals set the pace of neutrophil ageing in the circulation through TLR- and MyD88-dependent signaling, with germ-free and antibiotic-treated mice showing fewer aged neutrophils and less inflammation-driven organ damage (17).

In allogeneic hematopoietic stem cell transplantation (HSCT), the metabolite output of the intestinal microbiota tracks clinical outcome. Masetti et al. reviewed the microbiome-derived metabolites implicated in graft-versus-host disease (GVHD) and immune reconstitution after allografting (40), and Thiele Orberg et al. reported in transplant recipients that particular bacterial and bacteriophage consortia accompany protective intestinal metabolites and track with reduced GVHD and better survival (41). A causal counterpart has been shown for a single metabolite: desaminotyrosine protected mice against GVHD by promoting mTORC1- and STING-dependent intestinal regeneration (42). Comparable host–microbe relationships shape outcome in multiple myeloma and after stem cell transplantation more broadly (43). LPS, a component of the outer membrane of Gram-negative bacteria, translocates into the circulation when intestinal barrier integrity is lost, as occurs with ageing and chronic inflammation, and engages pattern recognition receptors on HSCs and niche cells; the resulting inflammatory signaling skews differentiation toward myeloid lineages. Cowan and Pietras have argued that this route links the intestinal microbiome to clonal hematopoiesis of indeterminate potential (CHIP) (44), and the wider case for the microbiome as a determinant of hematopoietic output and of the treatment of hematological disease has been set out by Fernandez Sanchez et al. (34).

At the level of the niche, microbial metabolites alter stromal cell function, the cytokine environment, and metabolic pathways such as sphingolipid and amino acid metabolism, and these changes converge on HSC fate. Lee et al. provided a defined example: lactate derived from lactic acid bacteria acts on GPR81 on leptin receptor-positive stromal cells and induces SCF, thereby promoting hematopoiesis and erythropoiesis (16). The converse is documented as well. In benzene-exposed mice, Wang et al. linked hematopoietic injury to loss of the genus unclassified_Muribaculaceae and to falls in butyrate and indole-3-propionic acid, tying a specific taxonomic shift to specific metabolite deficits and to impaired marrow function (11). Reduced microbial diversity after antibiotic treatment or during disease likewise lowers SCFA availability and delays hematopoietic recovery (18). These observations have motivated interventions along the same axis, including fecal microbiota transplantation, dietary strategies that raise SCFA production, and administration of individual metabolites such as desaminotyrosine (41, 42). Taken together, gut microbiota-derived metabolites and microbial components regulate HSC proliferation, differentiation bias and stress responsiveness through metabolic, epigenetic and TLR-mediated routes, shaping lineage bias and hematopoietic output in the bone marrow (**Table 1**).

**Table 1:** Gut microbiota–derived signals directly regulating bone marrow hematopoietic stem cell fate.

Gut-derived signal	Microbial source/feature	Primary sensing cells in bone marrow	Receptors/pathways involved	Effects on HSC fate and lineage bias	Impact on immune and bone homeostasis	Representative contexts	References
Butyrate	SCFA produced by fiber-fermenting anaerobes (e.g. Firmicutes)	HSCs, mesenchymal stromal cells, Tregs	HDAC inhibition; GPR41/GPR43	Maintains HSC quiescence; suppresses excessive myelopoiesis; supports balanced differentiation	Reduces inflammatory tone; indirectly limits osteoclastogenesis	Aging, antibiotic exposure, HSCT	(12–14)
Propionate/acetate	SCFAs from carbohydrate fermentation	HSCs, myeloid progenitors	GPR41/GPR43; metabolic reprogramming	Enhances emergency hematopoiesis under stress; promotes myeloid output	Supports innate immune readiness	Infection, chemotherapy-induced stress	(2, 10, 12)
Lactate	Produced by lactic acid bacteria	Leptin receptor^+^ stromal cells	GPR81 → SCF induction	Promotes HSC proliferation and erythropoiesis	Improves hematopoietic recovery	Anemia, microbiota-based therapy	(16)
Lipopolysaccharide (LPS)	Gram-negative bacteria	HSCs, macrophages, stromal cells	TLR4–MyD88–NF-κB	Drives myeloid-biased differentiation; reduces long-term HSC self-renewal	Sustains chronic inflammation; promotes osteoclast activation	Aging, dysbiosis, CHIP	(38, 44)
Bacterial CpG DNA	Commensal and pathogenic bacteria	HSCs, dendritic cells	TLR9	Modulates inflammatory signaling; preserves hematopoietic balance when controlled	Maintains bone mass and immune homeostasis	TLR9 deficiency models	(45)
Indole derivatives	Tryptophan metabolism by gut microbes	Stromal cells, immune cells	AhR signaling	Supports HSC stress resistance; modulates immune tone	Anti-inflammatory effects; protects niche	Toxic injury, metabolic stress	(11)
Microbial extracellular vesicles	Released by gut bacteria	Stromal cells, macrophages	PRRs, endocytic pathways	Alters niche cytokine environment	Shapes immune–bone crosstalk	Emerging mechanism	(106)

#### 2.1.2 Gut microbial signals regulate HSC fate via toll-like receptors

TLRs, particularly TLR9, serve as critical mediators linking gut microbial signals to the regulation of HSCs within the bone marrow. TLRs are pattern recognition receptors that detect microbe-associated molecular patterns (MAMPs) derived from the gut microbiota, thereby influencing immune responses and hematopoiesis. Among these, TLR9 has emerged as a pivotal receptor that modulates inflammation and bone metabolism by sensing bacterial DNA motifs. The absence of TLR9 disrupts this regulatory axis, leading to significant physiological consequences. Experimental evidence from TLR9-deficient (TLR9-/-) mice demonstrates that loss of TLR9 signaling results in gut microbiota dysbiosis, which triggers systemic low-grade chronic inflammation characterized by elevated pro-inflammatory cytokines such as tumor necrosis factor-alpha (TNFα), receptor activator of nuclear factor kappa-B ligand (RANKL), and interleukin-1 beta (IL-1β). This inflammatory environment promotes osteoclastogenesis, enhancing bone resorption and leading to notable bone loss. The dysbiotic gut microbiota in TLR9-/- mice acts as the primary driver of this systemic inflammation, highlighting the essential role of TLR9 in maintaining gut microbial homeostasis and preventing inflammatory bone disease (45).

Furthermore, single-cell RNA sequencing analyses of bone marrow cells from TLR9-/- mice reveal a myeloid-biased hematopoiesis, indicating that HSCs adapt to the chronic inflammatory environment by skewing differentiation toward the myeloid lineage. This myeloid bias not only exacerbates systemic inflammation but also contributes directly to increased osteoclast precursor populations, thereby linking altered hematopoietic output to bone pathology. These findings underscore the intricate crosstalk between gut microbial signals, TLR9-mediated immune regulation, and hematopoietic dynamics. The gut microbiota influences HSC fate decisions through TLR signaling pathways, and the disruption of TLR9 signaling impairs this regulatory feedback loop, resulting in pathological hematopoiesis and bone loss (45).

The broader context of the gut-bone marrow axis reveals that the gut microbiome modulates hematopoiesis through multiple metabolites and signaling molecules, including SCFAs, lactate, and bacterial extracellular vesicles, alongside TLR-mediated pathways. These microbial signals collectively influence HSC maintenance, differentiation, and immune cell production, which are crucial for systemic immune homeostasis and bone health. Aging further complicates this relationship by altering both the gut microbiota composition and hematopoietic function, potentially exacerbating dysregulation in TLR signaling and HSC fate control. Understanding how TLR9 integrates microbial cues to regulate inflammation and hematopoiesis may provide novel therapeutic targets to mitigate bone loss and hematopoietic disorders associated with gut dysbiosis and chronic inflammation (38).

#### 2.1.3 Single-cell sequencing reveals remodeling of bone marrow hematopoietic lineage

Single-cell RNA sequencing (scRNA-seq) technology has revolutionized the understanding of HSCs and their progeny within the bone marrow by enabling high-resolution profiling of cellular heterogeneity and lineage commitment dynamics (46). Under inflammatory or stress conditions, scRNA-seq studies have revealed a notable expansion of myeloid lineage cells and adaptive shifts in HSC states, reflecting the bone marrow’s capacity to remodel hematopoiesis in response to systemic signals (45, 47). For example, osteocyte ablation in mice, studied via scRNA-seq, demonstrated a mobilization of hematopoietic differentiation toward myeloid lineages, with expanded myeloid progenitors, neutrophils, and monocytes, accompanied by impaired lymphopoiesis and reduced B cells, indicating a senescence-associated secretory phenotype influencing lineage skewing (47). Similarly, in TLR9-deficient mice, single-cell transcriptomics uncovered myeloid-biased hematopoiesis linked to gut microbiota-induced systemic inflammation, highlighting the interplay between innate immune sensing, microbial signals, and hematopoietic remodeling (45). These findings underscore the plasticity of HSCs and progenitors to adapt their lineage output under inflammatory cues, which can be precisely dissected by scRNA-seq.

The application of scRNA-seq has also elucidated the molecular mechanisms governing lineage bias and adaptation. For instance, the regulation of lymphoid versus myeloid lineage commitment by mRNA decay factors Regnase-1 and Regnase-3 was characterized using single-cell transcriptomics, revealing control over myeloid-related gene expression and epigenetic landscapes in early HSPCs (48). Moreover, single-cell analyses have identified distinct HSC subpopulations with lineage priming, such as platelet-biased HSCs that increase with aging, providing insight into age-associated hematopoietic remodeling (49). These studies demonstrate that scRNA-seq enables the mapping of transcriptional trajectories and epigenetic states that underlie HSC adaptation to physiological and pathological stimuli.

Importantly, single-cell sequencing facilitates understanding of how gut-derived signals modulate bone marrow hematopoiesis. Dysbiosis and microbial metabolites influence systemic inflammation and hematopoietic output, as shown by altered immune cell populations and gene expression profiles in bone marrow under conditions like necrotizing enterocolitis and type 2 diabetes (50, 51). Single-cell approaches have revealed that gut microbiota-induced inflammatory signals can drive myeloid expansion and impair lymphoid progenitors, reshaping the bone marrow niche and hematopoietic lineage dynamics. Furthermore, the identification of stromal and endothelial niche cells by scRNA-seq has provided insights into how these microenvironmental components interact with HSCs and progenitors to regulate lineage specification and immune-metabolic homeostasis (46, 52).

Collectively, single-cell RNA sequencing has become an indispensable tool to dissect the complex cellular and molecular remodeling of bone marrow hematopoiesis under inflammatory states and microbial influences. It reveals adaptive expansion of myeloid cells, shifts in HSC lineage bias, and niche remodeling, thereby advancing understanding of how gut-derived signals and systemic inflammation reshape immune and bone metabolism. This knowledge lays the foundation for targeted interventions to modulate hematopoietic and immune dysfunction in diseases linked to gut microbiota and inflammation (**Figure 1**).

**Figure 1:**
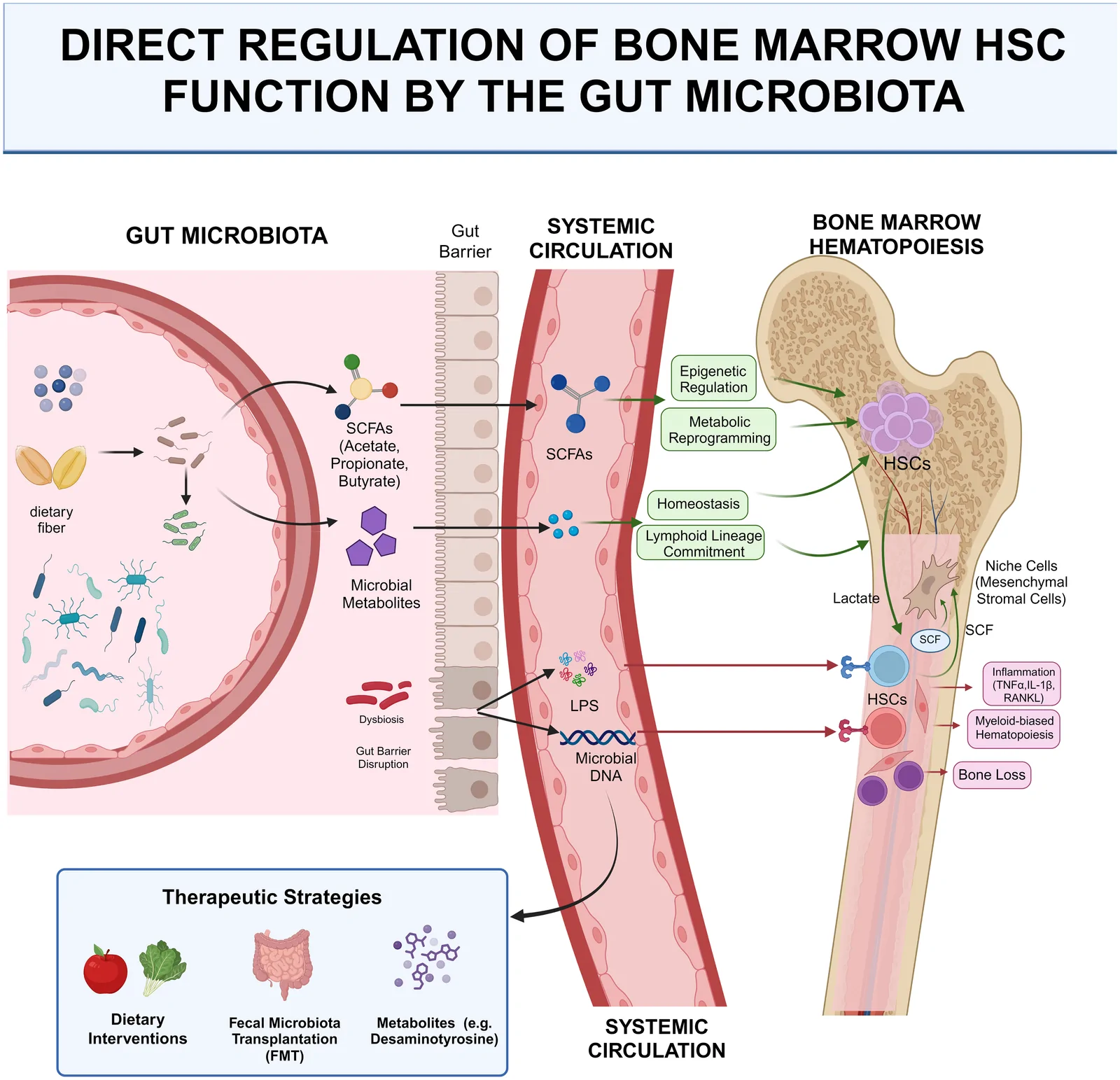
Direct regulation of bone marrow hematopoietic stem cell function by the gut microbiota. Dietary fiber fermented by the gut microbiota yields short-chain fatty acids (SCFAs; acetate, propionate and butyrate) and other microbial metabolites, which cross the intestinal barrier and reach the bone marrow through the systemic circulation, where they act on hematopoietic stem cells (HSCs) through epigenetic regulation, metabolic reprogramming, maintenance of homeostasis and lymphoid lineage commitment. Microbiota-derived lactate acts on niche cells (leptin receptor-positive mesenchymal stromal cells) to induce stem cell factor (SCF), supporting hematopoiesis and erythropoiesis. When the intestinal barrier is disrupted by dysbiosis, lipopolysaccharide (LPS) and bacterial DNA motifs translocate into the circulation and engage pattern recognition receptors on HSCs and niche cells, raising inflammatory tone (TNFa, IL-1b, RANKL), driving myeloid-biased hematopoiesis and promoting bone loss. Therapeutic strategies acting on this route include dietary interventions, fecal microbiota transplantation and administration of individual metabolites such as desaminotyrosine. Created in BioRender. (2026) https://BioRender.com/z8u6kyz.

### 2.2 Gut microbiota indirectly influences hematopoiesis by regulating the bone marrow microenvironment

#### 2.2.1 Bone marrow stromal cells and immune cell interactions

Bone marrow stromal cells (BMSCs), including mesenchymal stem/stromal cells (MSCs), osteoblasts, and macrophages, form a highly intricate and dynamic microenvironment that critically regulates HSC function and immune cell development. This stromal compartment provides essential structural support and secretes a variety of cytokines, chemokines, and extracellular vesicles that modulate HSC quiescence, self-renewal, and differentiation into diverse blood and immune lineages. MSCs, in particular, exhibit multilineage differentiation potential and immunomodulatory properties, influencing both innate and adaptive immune responses within the bone marrow niche (24, 53). Osteoblasts and osteoclasts contribute to the remodeling of the niche and regulate hematopoiesis through direct cell contacts and secretion of factors such as CXCL12, stem cell factor, and IL-7, which are crucial for lymphopoiesis and maintenance of memory T cells (54, 55). Macrophages and other immune cells residing in the bone marrow engage in bidirectional crosstalk with stromal cells, supporting tissue repair and maintaining homeostasis, while also shaping inflammatory responses that can impact hematopoiesis (24, 56). Recent single-cell transcriptomic studies have revealed the heterogeneity of stromal populations and their specialized roles in supporting hematopoietic and immune cell subsets, highlighting the complexity of the niche and its regulation (56, 57). Moreover, stromal cells release extracellular vesicles enriched with bioactive molecules that modulate immune cell function and hematopoietic progenitor fate, representing a critical mechanism of intercellular communication within the bone marrow microenvironment (58, 59). The interaction between BMSCs and immune cells is also implicated in pathological conditions such as leukemia, myelofibrosis, and bone metastases, where stromal remodeling and immune dysregulation contribute to disease progression and therapeutic resistance (24, 57, 60). Importantly, the gut microbiota indirectly influences this stromal-immune axis by modulating systemic inflammation and the secretion of factors that affect stromal cell function and hematopoiesis, thereby linking intestinal signals to bone marrow immune regulation (61). Collectively, these findings underscore the pivotal role of bone marrow stromal cells in orchestrating immune cell development and function through a complex network of cellular interactions and secreted mediators, which are modulated by both local and systemic signals including those derived from the gut microbiota. Multiple bone marrow niche components integrate gut-derived metabolic, inflammatory, and neuroendocrine signals to coordinately regulate hematopoietic stem cell maintenance, immune cell differentiation, and bone remodeling (**Table 2**). Li et al. gave a concrete example of this crosstalk in the ageing marrow, showing that senescent immune cells release grancalcin, which acts on mesenchymal progenitors and accelerates skeletal ageing (59), and He et al. showed that a bone–liver circuit operating through a Pinch–CXCL12–MBL2 axis shapes both immune and hematopoietic output (60), indicating that the niche integrates signals arriving from distant organs as well as from the gut.

**Table 2:** Bone marrow niche components integrating gut microbiota signals.

Bone marrow component	Key gut-regulated inputs	Major signaling mediators	Functional effects on hematopoiesis	Contribution to immune balance	Relevance to bone metabolism	References
Mesenchymal stromal cells (MSCs)	SCFAs, lactate, inflammatory cytokines	CXCL12, SCF, IL-7	Maintain HSC retention and quiescence; support lymphopoiesis	Immunomodulation; T cell support	Regulate osteoblast–osteoclast balance	(24, 53, 54)
Osteoblasts	Systemic inflammation; TLR ligands	RANKL, OPG	Influence HSC niche localization	Link immunity to bone remodeling	Control bone formation	(54, 55, 61)
Osteoclasts	TNFα, IL-1β, RANKL induced by dysbiosis	NF-κB, MAPK	Indirectly affect niche integrity	Promote inflammatory microenvironment	Drive bone resorption	(45, 61)
Bone marrow macrophages	LPS, microbial metabolites	TLRs, cytokines	Regulate HSC dormancy and mobilization	Amplify or suppress inflammation	Influence bone turnover	(20, 24)
Endothelial cells	SCFAs, neuroendocrine signals	Notch, VEGF	Support HSC trafficking	Coordinate immune cell entry/exit	Maintain vascular niche	(19, 54)
Bone marrow adipocytes	Metabolic inflammation, aging-associated dysbiosis	Adipokines, IL-6	Suppress lymphoid output; favor myelopoiesis	Promote inflammaging	Negatively affect bone quality	(25, 26, 67, 68)
Neural inputs (SNS/vagus)	Microbial metabolites, gut–brain signaling	β-adrenergic, neuropeptides	Regulate circadian HSC release	Shape immune tone	Modulate osteoclast activity	(27, 65, 69)

#### 2.2.2 Regulation of systemic inflammation and bone marrow immune homeostasis

Systemic inflammation induced by gut microbiota dysbiosis exerts profound effects on bone marrow immune cell activation and function, reshaping hematopoiesis and immune homeostasis. Dysbiosis, characterized by an imbalance in microbial communities, triggers chronic low-grade systemic inflammation that activates bone marrow immune cells, leading to altered differentiation and proliferation patterns. For instance, Toll-like receptor 9 (TLR9) deficiency causes gut microbiota dysbiosis, which in turn induces systemic chronic inflammation marked by elevated pro-inflammatory cytokines such as tumor necrosis factor-alpha (TNFα), RANKL, and interleukin-1β (IL-1β). These cytokines promote osteoclastogenesis, resulting in bone loss, and simultaneously drive myeloid-biased hematopoiesis by adapting HSCs to the inflammatory environment, thus perturbing immune cell homeostasis in the bone marrow (45). Similarly, obesity-related metabolic inflammation alters the bone marrow microenvironment by increasing inflammatory cytokines, which skew HSC differentiation toward myeloid progenitors and impair bone integrity and immune function. This remodeling of the bone marrow niche compromises immune cell functions and exacerbates systemic inflammation, highlighting the bidirectional relationship between metabolic inflammation and bone marrow hematopoiesis (62, 63). Chronic inflammatory conditions such as cardiometabolic diseases also dysregulate the bone marrow niche, disrupting the balance between HSC self-renewal and differentiation. This leads to enhanced myelopoiesis and the excessive production of pro-inflammatory myeloid cells, which further fuel systemic inflammation and disease progression. Key inflammatory mediators including TNFα and RANKL not only stimulate bone resorption by activating osteoclasts but also promote expansion of myeloid cells, thereby linking bone metabolism with immune dysregulation (64). The sympathetic nervous system (SNS) modulates bone marrow immune cell dynamics by regulating β-adrenoreceptor signaling; prolonged activation of this pathway under inflammatory states such as hypertension leads to immune dysregulation and sustained neuroinflammation, further aggravating systemic inflammation and bone marrow immune imbalance (65). Experimental models of traumatic brain injury (TBI) reveal that systemic inflammation intensifies T cell-mediated graft-versus-host disease and causes regional bone marrow hematopoietic cell depletion, which can be ameliorated by mesenchymal stromal cell therapy, underscoring the sensitivity of bone marrow immune homeostasis to systemic inflammatory insults (66). Moreover, inflammatory cytokines such as IL-1β activate signaling pathways like p38/MAPK in HSCs within the bone marrow niche, biasing their differentiation toward myeloid lineages and perpetuating chronic inflammation, as demonstrated in obesity models (63). The interplay between systemic inflammatory mediators and bone marrow stromal cells creates a microenvironment conducive to myeloid cell expansion and osteoclast activation, thereby linking systemic inflammation to bone loss and immune imbalance. Additionally, bone marrow adipose tissue (BMAT), which increases with age and metabolic disturbances, secretes adipokines and pro-inflammatory cytokines that contribute to low-grade systemic inflammation (inflammaging), further disrupting hematopoietic and immune homeostasis in the bone marrow (67, 68). Collectively, these findings illustrate that gut microbiota dysbiosis-induced systemic inflammation activates bone marrow immune cells and alters their function through inflammatory cytokines such as TNFα and RANKL. This promotes osteoclastogenesis and myeloid cell proliferation, thereby affecting bone metabolism and immune balance. Understanding these mechanisms provides insight into the gut-bone marrow axis as a critical regulator of systemic inflammatory states and offers potential therapeutic targets for modulating bone marrow immune homeostasis and preventing inflammation-induced bone and immune disorders.

#### 2.2.3 Microbiota-mediated neuro-immune regulatory mechanisms

The gut-brain-bone marrow axis represents a complex, bidirectional communication network where neural signaling plays a pivotal role in modulating bone marrow hematopoiesis and immune homeostasis. Emerging evidence indicates that neural circuits, particularly those involving the vagus nerve and enteric nervous system, integrate signals from the gut microbiota to influence bone marrow function. For instance, sensory neurons detect microbial metabolites and relay this information centrally, which in turn modulates autonomic outputs to the bone marrow niche. This neural regulation affects hematopoietic stem and progenitor cells (HSPCs) proliferation, differentiation, and mobilization, thereby shaping systemic immune responses. The amygdala-vagal-glandular circuit exemplifies such neuroimmune crosstalk, where central nervous system activity influences intestinal microbial populations through vagal pathways, indirectly impacting peripheral immune compartments including the bone marrow (69). Moreover, alterations in gut microbiota composition can modify neural signaling pathways that regulate bone marrow microenvironments, thus affecting the balance between myeloid and lymphoid lineage commitment. This axis is crucial for maintaining immune surveillance and adapting hematopoiesis in response to environmental cues. Disruptions in neural inputs, as observed in neuropathic pain or spinal cord injury models, lead to gut dysbiosis and altered immune cell profiles in the bone marrow, underscoring the importance of intact neuro-immune communication for hematopoietic regulation (70). Collectively, these findings underscore that neural signaling within the gut-brain-bone marrow axis is a fundamental mechanism by which the microbiota orchestrates hematopoietic and immune states, enabling adaptive responses to maintain systemic homeostasis.

Gut microbiota exert profound effects on the bone marrow microenvironment by modulating the production and availability of neurotransmitters and hormones that influence hematopoiesis and immune function. Microbial metabolites, such as SCFAs, tryptophan derivatives, and neuroactive compounds including serotonin and gamma-aminobutyric acid (GABA), serve as key mediators in this neuroimmune dialogue. These molecules can cross the intestinal barrier and interact with receptors on enteric neurons, immune cells, and bone marrow stromal cells, thereby shaping the hematopoietic niche. For example, serotonin synthesized by gut microbes or enterochromaffin cells modulates hematopoietic stem cell proliferation and mobilization, linking microbial activity to systemic immune readiness (71). Additionally, microbial regulation of neurotransmitter systems influences the hypothalamic-pituitary-adrenal (HPA) axis, which controls circulating hormone levels such as cortisol, impacting bone marrow immune cell differentiation and function (72). The neuropeptide calcitonin gene-related peptide (CGRP), produced by sensory neurons responsive to microbial cues, directly modulates T lymphocyte activation and cytokine production, fine-tuning adaptive immune responses (73). Furthermore, gut microbiota-driven modulation of the enteric nervous system affects intestinal epithelial and immune cell interactions, which indirectly influence systemic immune homeostasis and bone marrow activity (74). These neuroendocrine and neurotransmitter-mediated pathways enable the bone marrow microenvironment to adapt dynamically to changes in microbial composition and host physiological states, ensuring appropriate hematopoietic output and immune defense. Therapeutic strategies targeting these microbiota-neuroimmune axes, such as probiotics, fecal microbiota transplantation, and neuromodulation, hold promise for correcting hematopoietic and immune dysregulation in various diseases (75). Thus, gut microbiota-mediated regulation through neurotransmitters and hormones constitutes a critical mechanism for adaptive hematopoietic and immune adjustments within the bone marrow niche (**Figure 2**).

**Figure 2:**
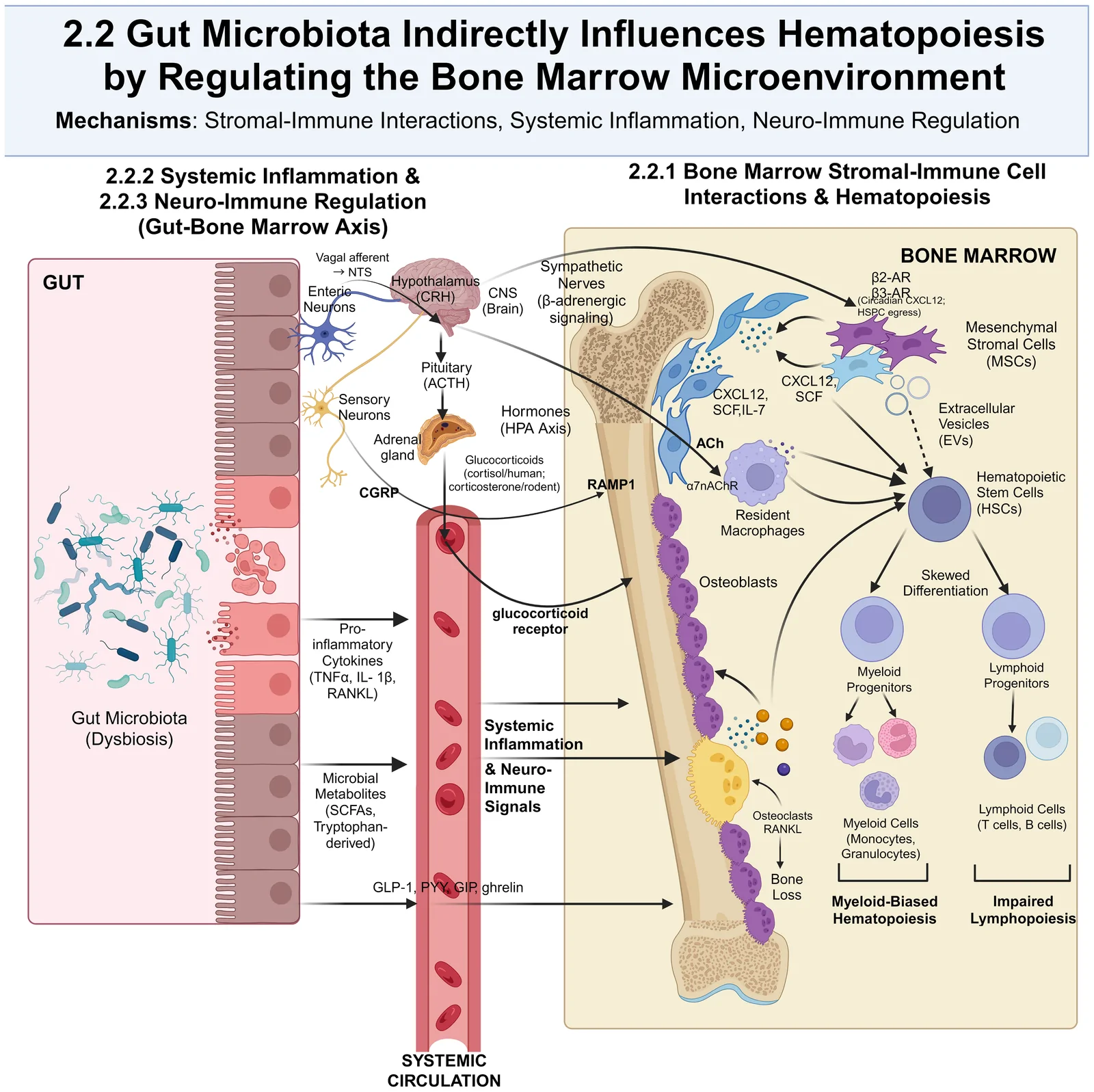
Indirect regulation of bone marrow immune homeostasis through the gut–neuro–immune axis. Gut microbiota indirectly regulates bone marrow hematopoiesis and immune homeostasis through integrated neuro-immune, neuroendocrine and stromal pathways. Microbial metabolites, including SCFAs, tryptophan derivatives, serotonin (5-HT) and γ-aminobutyric acid (GABA), modulate enteric and central nervous system signaling via the vagus nerve and autonomic circuits. The neuro-immune signals conveyed to the marrow are indicated: vagal afferent input to the nucleus tractus solitarius and efferent cholinergic output acting through the α7 nicotinic acetylcholine receptor (α7nAChR) on marrow macrophages, that is, the cholinergic anti-inflammatory pathway; sympathetic noradrenergic fibers signaling through β2- and β3-adrenergic receptors on mesenchymal stromal cells and osteoblasts, which set circadian CXCL12 expression and HSPC egress; and calcitonin gene-related peptide (CGRP) released from nociceptive sensory fibers, acting on RAMP1 on immune and stromal cells. Hypothalamic–pituitary–adrenal (HPA) axis hormones are shown along the same route: hypothalamic corticotropin-releasing hormone (CRH) drives pituitary adrenocorticotropic hormone (ACTH), which drives adrenal glucocorticoid release — cortisol in humans, corticosterone in rodents — acting on the glucocorticoid receptor of stromal and immune cells. Enteroendocrine hormones whose secretion is microbially regulated, namely GLP-1, PYY, GIP and ghrelin, are shown as a parallel humoral route from gut to bone and marrow. These inputs converge on the bone marrow microenvironment, influencing mesenchymal stromal cells, osteoblasts, macrophages, endothelial cells, and immune populations. Systemic inflammatory mediators and neuroendocrine hormones further reshape cytokine gradients, niche architecture, and immune cell trafficking, collectively affecting hematopoietic stem cell quiescence, proliferation, and lineage commitment. Created in BioRender. (2026) https://BioRender.com/z8u6kyz.

Beyond serotonin and GABA, the intestine releases a family of enteroendocrine hormones whose secretion is under microbial control and whose receptors are present on bone and immune cells; this arm of the axis has been examined unevenly. SCFAs are a direct secretagogue: propionate and other SCFAs stimulate glucagon-like peptide-1 (GLP-1) release from intestinal L cells through FFAR2, which places GLP-1 and the co-secreted peptide YY (PYY) downstream of fiber fermentation (76). Their skeletal effects are, in outline, opposed. GLP-1, glucagon-like peptide-2 and glucose-dependent insulinotropic polypeptide (GIP) inhibit bone resorption, and GIP additionally promotes bone formation, whereas PYY favors resorption, so a shift in the microbial fiber supply is transmitted to bone turnover through more than one hormone at once (77, 78). Ghrelin, whose circulating concentration is likewise sensitive to microbial composition, acts on the lymphoid side: in mice it protected thymic epithelium from conditioning-regimen damage and accelerated CD4+ T cell recovery after bone marrow transplantation, one of the few instances in which a gut hormone has been tested directly against a marrow-relevant immune endpoint (79). Vasoactive intestinal peptide (VIP), released by enteric and marrow-associated neurons, shapes T cell responses and graft-versus-leukemia activity after allogeneic transplantation, and its pharmacological blockade enhances anti-leukemic T cell immunity (80); whether microbial cues set VIP tone within the marrow itself has not been tested. For cholecystokinin and resistin the position is weaker again: to our knowledge no study has followed a microbiota-driven change in either hormone through to a hematopoietic or marrow immune readout, and they remain an untested part of this route. That unevenness is itself informative, and closing it — by measuring the enteroendocrine profile alongside marrow phenotype in the same animals — is among the more tractable gaps in the field.

### 2.3 Development of gut-associated B cells and formation of immune memory in early life

#### 2.3.1 Neonatal time window of B cell lineage

Emerging research has delineated a critical neonatal time window during which a significant portion of the adult B cell repertoire is established, highlighting the unique ontogeny and functional specialization of these early-life-derived B cells. Lineage tracing studies in murine models have revealed that many adult B cells, including B-1a cells and clonally related IgA plasma cells residing in the gut and bone marrow, originate predominantly within a restricted neonatal period (81). These neonatal B cells form a self-sustaining layer that actively contributes to steady-state IgA responses, which are essential for maintaining intestinal mucosal immunity and homeostasis. Notably, these IgA plasma cells demonstrate reactivity to commensal microbiota and are maintained by B cell memory within chronic germinal centers of the gut, indicating a durable imprinting of neonatal B cell responses that persist into adulthood (81). Furthermore, neonatal infections, such as rotavirus, recruit distinct IgA clones compared to those generated during adult infection, underscoring the unique immunological imprinting during this early life window (81). Complementing these findings, temporally controlled lineage tracking using a RAG2-based system has confirmed that B-1a cells generated during embryonic and neonatal periods dominate the adult B-1a compartment across multiple anatomical sites including bone marrow, peritoneal cavity, and spleen (82). These early-life B-1a cells exhibit a restricted B cell receptor (BCR) repertoire that diversifies progressively through neonatal development into adulthood, suggesting a maturation and adaptation process that shapes the adult B-1a pool (82). Importantly, more than half of plasmablasts and plasma cells in adult bone marrow bear a history of embryonic and neonatal RAG2 expression, indicating a shared origin with B-1a cells and highlighting the contribution of early-life hematopoiesis to the adult humoral compartment (82). The layered immune system hypothesis further supports this concept by proposing that distinct waves of hematopoiesis during fetal and neonatal development generate unique immune cell subsets, including B-1a progenitors, which are temporally restricted and not replenished by adult HSCs (83). This developmental layering results in a complex immune architecture where neonatal-derived B cells provide specialized functions, particularly in mucosal immunity. Functionally, these neonatal B-1a cells and IgA plasma cells are critical for maintaining intestinal barrier integrity and regulating microbiota composition, thereby contributing to immune tolerance and protection against pathogens (81). Transcriptional regulation also plays a pivotal role; for instance, the transcriptional repressor Bach2 is essential for the maintenance and self-renewal of mature B-1 cells in peripheral niches, although it does not affect the initial development of B-1 progenitors during fetal and neonatal stages (84). Additionally, factors such as capicua (CIC) modulate postnatal B-1a cell development and survival by regulating B cell receptor signaling pathways, thereby balancing B-1 versus conventional B-2 cell formation (85). Collectively, these findings underscore a defined neonatal window during which B cell lineages, particularly B-1a cells and IgA-secreting plasma cells, are generated and imprinted with unique functional properties that shape lifelong immune responses, especially within the gut mucosal environment. Understanding this temporal and developmental specificity offers critical insights into how early-life immune programming influences adult immunity and may inform strategies to manipulate these responses for improved mucosal vaccine design and treatment of immune-mediated diseases.

#### 2.3.2 The connection between intestinal IgA plasma cells and bone marrow B cells

IgA plasma cells (PCs) are a specialized subset of antibody-secreting cells that play a pivotal role in mucosal immunity, particularly within the gut, but they are also found in systemic compartments such as the bone marrow. Both intestinal and bone marrow IgA PCs originate from a common hematopoietic progenitor pool, reflecting a shared ontogeny that ties mucosal and systemic humoral immunity together. Lineage tracing studies in murine models have demonstrated that a significant portion of adult IgA PCs, including those residing in the gut and bone marrow, derive from neonatal hematopoiesis, indicating that early-life B cell progenitors give rise to a self-sustaining population of IgA-secreting cells that persist into adulthood (81). This developmental link is further supported by clonal analyses showing shared IgA clonotypes between the gut lamina propria and bone marrow, suggesting that these cells, while tissue-resident and non-recirculating, emerge from the same B cell lineage and maintain related antigen specificities (86).

Within the gut, IgA PCs are generated predominantly in chronic germinal centers located in Peyer’s patches and isolated lymphoid follicles, which serve as inductive sites where B cells undergo somatic hypermutation and class-switch recombination to IgA. These germinal centers are sustained by continuous antigenic stimulation from the commensal microbiota, enabling the production of IgA antibodies that are specific to the resident microbial community and contribute to intestinal homeostasis (81, 87). The chronic nature of these germinal centers ensures a steady-state production of IgA PCs that migrate to the lamina propria and secrete polymeric IgA, which is then transported across the epithelium to the mucosal surface. This mucosal IgA plays a dual role in immune defense by neutralizing pathogens and maintaining a balanced relationship with commensal bacteria (87).

Interestingly, IgA PCs are not confined to the mucosal environment. A considerable population of IgA PCs resides in the bone marrow, where they contribute to systemic IgA levels. These bone marrow IgA PCs are long-lived and exhibit distinct transcriptional programs compared to their gut counterparts, reflecting tissue-specific adaptation. For example, bone marrow IgA PCs show increased mitochondrial dependency and secrete higher amounts of antibodies per cell compared to gut IgA PCs, which rely more on glycolytic metabolism and secrete fewer antibodies individually (86, 88). Despite these functional differences, the clonal relationship between gut and bone marrow IgA PCs indicates a shared origin and suggests that gut-derived IgA PCs can seed the bone marrow plasma cell niches, thereby linking mucosal immune responses to systemic humoral immunity (86).

The maintenance of IgA PC populations in both gut and bone marrow is tightly regulated by chemokine receptors and adhesion molecules that control their localization. For instance, the transcription factor Krüppel-like factor 2 (KLF2) orchestrates the compartmentalization of IgA PCs by modulating expression of CCR9, integrins, and sphingosine-1-phosphate receptors, which govern trafficking between mucosal sites, lymphoid organs, and the bone marrow. Loss of KLF2 disrupts IgA PC distribution, leading to their accumulation in mesenteric lymph nodes and Peyer’s patches, and a marked reduction in IgA PCs in the bone marrow and intestinal lamina propria, ultimately impairing IgA responses to enteric antigens (89). This highlights the importance of coordinated migration and localization signals in sustaining effective IgA-mediated immunity across different tissue compartments.

Moreover, the antigen specificity of IgA antibodies produced by gut and systemic PCs can differ, reflecting tissue-specific immune imprinting. Studies examining responses to bacterial antigens such as those from Bacteroides fragilis and pathogenic Escherichia coli reveal that systemic IgA responses require robust intestinal colonization and Peyer’s patch involvement, whereas mucosal IgA may target distinct epitopes and glycosylation patterns not fully mirrored in serum IgA (90, 91). This compartmentalization of IgA specificity suggests that while gut and bone marrow IgA PCs share developmental origins, their functional roles and antigenic targets may be tailored to their local microenvironments.

In summary, intestinal IgA plasma cells and bone marrow B cells are intimately connected through a shared hematopoietic origin and clonal relationships established in gut-associated lymphoid tissues. Chronic germinal centers in the intestine sustain IgA PC generation and specificity toward commensal microbiota, while a subset of these cells migrates to and resides in the bone marrow, contributing to systemic IgA pools. The compartmentalization and functional specialization of IgA PCs in these tissues are governed by transcriptional programs and migratory cues, underscoring the dynamic interplay between mucosal and systemic humoral immunity. Understanding these connections provides valuable insights into how gut-derived immune signals reshape hematopoiesis and systemic immune status, with implications for vaccine design and treatment of immune-mediated diseases.

#### 2.3.3 Long-term effects of early-life infections on B cell lineage

Early-life infections, particularly during the neonatal period, have a profound and lasting impact on the B cell lineage, shaping immune memory and antibody specificity well into adulthood. Neonatal infections such as rotavirus induce the expansion of specific IgA clones that are distinct from those generated by the same pathogen in adults, indicating a unique imprinting of the B cell repertoire during this critical developmental window. This phenomenon is exemplified by the findings that a substantial portion of adult B cells, including IgA plasma cells in the gut and bone marrow, originate from a restricted neonatal period and are maintained through self-sustaining memory within chronic germinal centers in the gut. These early-life derived B cells exhibit clonal interrelatedness and specificity toward commensal microbiota, suggesting that neonatal microbial exposures intricately shape the mucosal immune landscape and antibody repertoire in adulthood (81). The distinct nature of neonatal IgA clones compared to those induced in adults underscores the importance of the timing of antigen exposure in determining long-term humoral immunity.

Moreover, the early gut microbiota and infection history not only influence the clonal composition of B cells but also modulate their functional differentiation pathways. For instance, micronutrients such as vitamin C have been shown to enhance antibody responses by facilitating IL-21/STAT3-dependent plasma cell differentiation via epigenetic remodeling mechanisms. Vitamin C acts as a cofactor for TET enzymes that mediate DNA demethylation at key loci controlling plasma cell fate, thereby potentiating efficient antibody production. This highlights how early nutritional and microbial signals can epigenetically prime B cells for robust antibody responses, potentially affecting the quality and durability of humoral immunity established in early life (92). These epigenetic modifications may serve as a molecular memory of early environmental exposures, influencing B cell lineage commitment and function long-term.

The long-term consequences of early infections are not limited to antibody specificity but extend to the overall immune competence and susceptibility to infections later in life. While the immune system undergoes physiological aging, characterized by telomeric shortening and altered lymphocyte phenotypes, early-life infection history and the resultant shaping of B cell memory may modulate these aging processes. For example, allogeneic hematopoietic cell transplant recipients exhibit some aging-like features in their lymphocytes, including B cells, which may be influenced by prior infection history and immune challenges. This suggests that the early-life immune imprinting could have implications for immune resilience and the capacity to respond to infections or immunotherapies in adulthood (93). Understanding how neonatal infections and microbiota exposures program the B cell compartment provides critical insights into the developmental origins of immune memory and offers potential avenues for interventions aimed at optimizing lifelong immunity (**Figure 3**).

**Figure 3:**
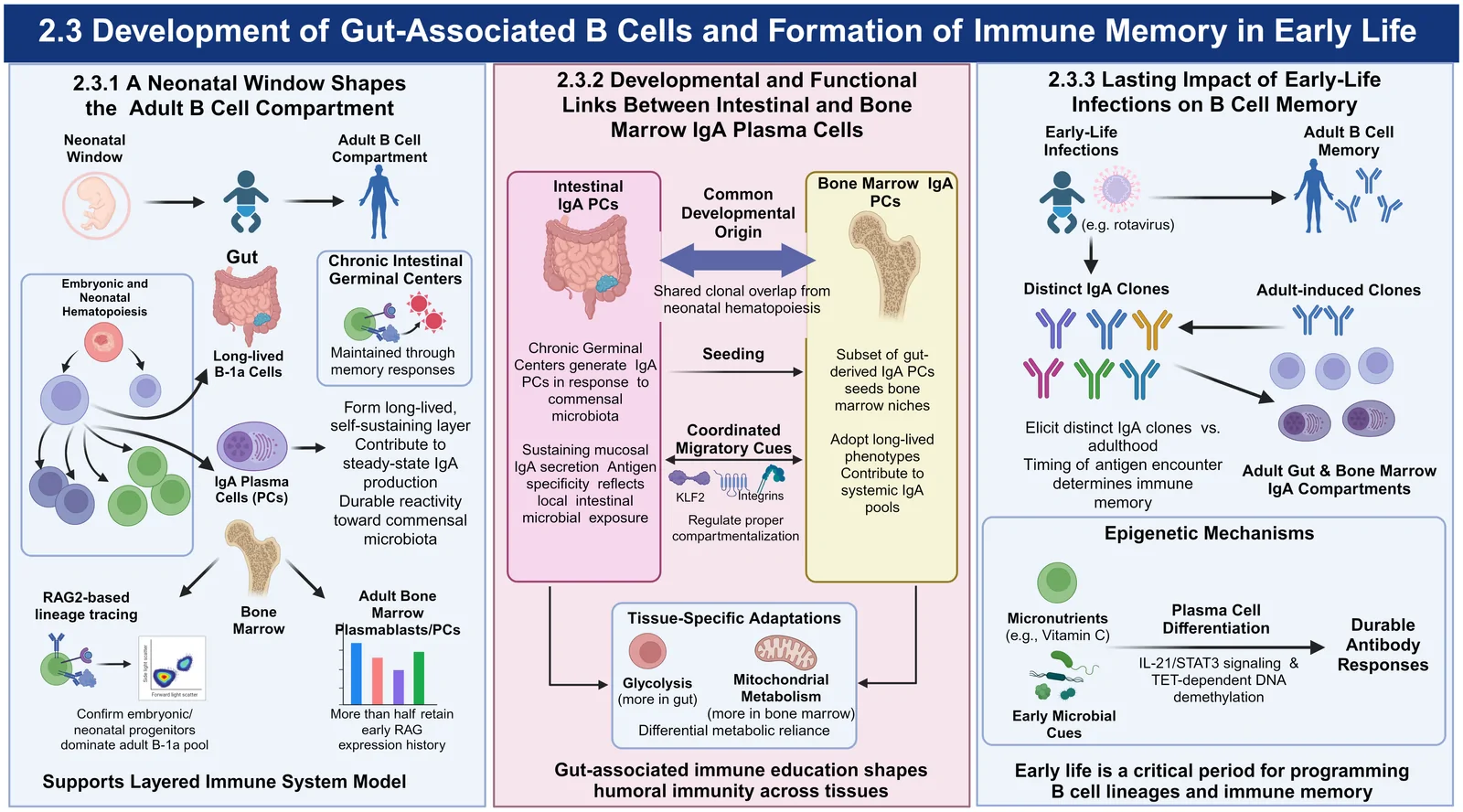
Early-life gut microbiota imprinting of B cell lineage and long-term immune memory. Early-life exposure to gut microbiota establishes a critical developmental window during which distinct B cell lineages and long-lived immune memory are formed. Neonatal waves of hematopoiesis generate B-1a cells and IgA-secreting plasma cells that persist into adulthood and contribute to steady-state mucosal and systemic immunity. Chronic antigenic stimulation by commensal microbiota in gut-associated lymphoid tissues shapes durable IgA repertoires and clonal relationships between intestinal and bone marrow plasma cells. Early-life infections and microbial colonization imprint long-lasting transcriptional and epigenetic programs that determine B cell fate, antibody specificity, and immune memory throughout life. Created in BioRender. (2026) https://BioRender.com/z8u6kyz.

### 2.4 Clinical significance and potential therapeutic strategies of the gut microbiota-bone marrow axis

#### 2.4.1 Immune-related diseases and dysregulation of the gut-bone marrow axis

The dysregulation of the gut-bone marrow axis has emerged as a pivotal factor in the pathogenesis of various immune-related diseases, including osteoporosis and autoimmune disorders. Gut microbiota imbalance, or dysbiosis, is closely linked to bone metabolic diseases such as glucocorticoid-associated osteonecrosis of the femoral head (GA-ONFH) and systemic autoimmune conditions. In GA-ONFH, for instance, alterations in gut microbial composition lead to decreased levels of key metabolites like butyric acid, a short-chain fatty acid (SCFA) crucial for maintaining bone marrow mesenchymal stem cell (BMSC) function and modulating inflammatory cytokines such as TNF-α, IL-2, and IL-4. Experimental depletion of gut microbiota exacerbates bone damage, while fecal microbiota transplantation (FMT) restores butyric acid levels and ameliorates disease progression by improving the local inflammatory environment in bone tissue (94). This exemplifies how gut microbial metabolites influence bone marrow immune cell behavior and bone homeostasis.

Autoimmune diseases such as type 1 diabetes (T1D) also illustrate the gut-bone marrow axis’s role in disease progression. Deficiency of G-protein-coupled receptor 41 (GPR41), a receptor for SCFAs produced by gut microbes, aggravates T1D by enhancing dendritic cell (DC) maturation and promoting gut immune dysregulation. The increased migration of gut-primed IFN-γ+ T cells to the pancreas in GPR41-deficient mice underscores the systemic immune consequences of gut microbiota imbalance and its impact on bone marrow-derived immune precursors (15). Similarly, in inflammatory bowel disease (IBD), dysbiosis leads to systemic inflammation and altered bone marrow hematopoiesis, contributing to chronic intestinal inflammation and immune dysregulation. For example, partial recombination-activating gene deficiency (pRD) results in colitis characterized by Th1/Th17 inflammatory signatures and microbial depletion of SCFA-producing species, which impairs peripheral Treg induction and exacerbates gut inflammation. Bone marrow transplantation, rather than antibiotics or biologics, effectively restores immune and microbial homeostasis, highlighting the centrality of bone marrow immune regulation in gut inflammatory diseases (95).

The systemic inflammation associated with gut microbiota dysbiosis extends to bone health, where immune-mediated mechanisms drive bone loss and remodeling disorders. Dysregulated immune responses involving RANKL and IL-17 hyperactivity promote osteoclastogenesis and osteoporosis. The bone marrow microenvironment serves as a reservoir for immune cells that are shaped by gut-derived signals, including microbial metabolites that modulate epigenetic and receptor-mediated pathways influencing osteoimmune interactions (61). Furthermore, gut microbial translocation and systemic inflammation induce trained myelopoiesis in the bone marrow, which can exacerbate bone-destructive diseases such as periodontitis and hematologic malignancies, including multiple myeloma and leukemia. In these conditions, microbial dysbiosis and impaired intestinal barriers facilitate migration of microbial components and immune cells to the bone marrow, reshaping the local immune microenvironment and promoting tumorigenesis or bone pathology (96–98).

The same logic extends to the solid tumors that marrow-derived effectors must control: in esophageal squamous cell carcinoma, gut Ligilactobacillus salivarius and its tryptophan metabolite indole-3-lactic acid engage the aryl hydrocarbon receptor to suppress NF-κB signaling in NKG7+CD8+ precursor-exhausted T cells and blunt the response to anti-PD-1 therapy, so that a single microbial metabolite sets the functional state of an effector population generated in the bone marrow (99).

Collectively, these findings underscore that systemic inflammation and bone marrow hematopoietic skewing are key mechanisms by which gut microbiota dysbiosis contributes to immune-related diseases. The gut-bone marrow axis represents a dynamic immunometabolic interface where microbial signals regulate hematopoiesis, immune cell differentiation, and inflammatory responses that influence disease onset and progression. Therapeutic interventions targeting this axis, such as microbiota modulation, metabolite supplementation, and bone marrow transplantation, hold promise for restoring immune homeostasis and mitigating disease severity in osteoporosis, autoimmune disorders, and inflammatory bowel diseases (15, 61, 94–96). Dysregulation of the gut microbiota–bone marrow axis is implicated in a broad spectrum of immune-related and bone metabolic disorders, highlighting emerging microbiota-targeted therapeutic strategies for restoring hematopoietic and immune homeostasis (**Table 3**).

**Table 3:** Clinical disorders linked to dysregulation of the gut microbiota–bone marrow axis and therapeutic opportunities.

Disease/condition	Gut microbiota alteration	Bone marrow hematopoietic phenotype	Key immune mediators	Bone-related consequences	Microbiota-targeted interventions	References
Osteoporosis	Loss of SCFA-producing bacteria	Myeloid-biased hematopoiesis	TNFα, RANKL, IL-17	Increased osteoclastogenesis	Probiotics, SCFA supplementation	(39, 61)
Autoimmune diseases (e.g. T1D)	Reduced microbial diversity	Enhanced inflammatory DC and T cell output	IFNγ, IL-1β	Bone loss secondary to inflammation	Prebiotics, receptor targeting (GPR41)	(15)
Inflammatory bowel disease	Dysbiosis with barrier disruption	Trained myelopoiesis	Th1/Th17 cytokines	Osteopenia	FMT, immune–microbiota co-therapy	(95)
Aging/inflammaging	Reduced microbial resilience	Clonal hematopoiesis; myeloid skewing	Chronic low-grade inflammation	Reduced bone mass	Microbiota rejuvenation strategies	(38, 67, 100)
Hematologic malignancies	Microbial translocation	Aberrant HSC differentiation	NF-κB activation	Bone marrow niche remodeling	Adjunct microbiota modulation	(28, 96, 98)
Chemotherapy-induced myelosuppression	Antibiotic-associated dysbiosis	Impaired HSC recovery	Reduced IFN-I signaling	Delayed bone marrow regeneration	Probiotics, metabolite replacement	(18, 101)
HSCT complications (GVHD)	Loss of protective metabolites	Dysregulated immune reconstitution	Excessive inflammation	Secondary bone loss	Targeted FMT, metabolite therapy	(40–42)

#### 2.4.2 Therapeutic prospects of targeting gut microbiota to regulate hematopoiesis

Targeting the gut microbiota to regulate hematopoiesis represents a promising therapeutic frontier, leveraging interventions such as probiotics, microbial ecosystem modulators, and microbial metabolites to restore bone marrow hematopoietic homeostasis. Probiotic administration and microbial metabolite supplementation have been shown to beneficially modulate the gut microbial community, which in turn influences HSC function and immune cell development. For instance, oral administration of lactic acid-producing bacteria (LAB) activates SCF secretion from leptin receptor-positive bone marrow mesenchymal stromal cells, thereby enhancing hematopoiesis and erythropoiesis in a Gpr81-dependent manner, highlighting the direct impact of microbiota-derived metabolites on hematopoietic niche cells (16). Similarly, FMT from young to aged mice rejuvenates aged HSCs by suppressing inflammatory signaling and promoting lymphoid differentiation, demonstrating the potential of microbiota modulation to reverse age-associated hematopoietic decline (100). In immunosuppressed models induced by cyclophosphamide, protopanaxadiol (PPD) enhances bone marrow hematopoiesis and immunity by restoring gut microbiota diversity and increasing beneficial bacteria such as Lactobacillus, while reducing pathogenic Escherichia-Shigella; notably, depletion of gut microbiota abolishes these hematopoietic benefits, underscoring the microbiota’s essential role (101). Moreover, traditional Chinese medicine formulations like Siwu paste improve hematopoiesis in anemia rats by modulating gut microbial composition and metabolites, particularly amino acids and lipids, which correlate positively with hematopoietic efficacy (102). These findings collectively support the concept that microbiota-targeted therapies can restore hematopoietic balance by reshaping microbial communities and their metabolic outputs.

Concurrently, regulation of TLRs and associated signaling pathways offers another therapeutic avenue to suppress inflammation and promote bone metabolism balance, thereby indirectly supporting hematopoiesis. TLR9 deficiency models reveal that loss of TLR9 signaling leads to gut microbiota dysbiosis, systemic chronic inflammation, and low bone mass due to enhanced osteoclastogenesis driven by elevated inflammatory cytokines such as TNFα, RANKL, and IL-1β. This inflammatory environment also skews hematopoiesis toward myeloid-biased differentiation, linking TLR signaling with both immune and bone homeostasis (45). The interplay between gut microbiota and TLR pathways is critical, as microbial dysbiosis can trigger aberrant TLR activation, perpetuating inflammation and disrupting hematopoietic and bone remodeling processes. Therapeutic strategies aimed at modulating TLRs or their downstream effectors could therefore attenuate inflammation-induced hematopoietic dysfunction and osteoclastic bone loss. For example, targeting microbial metabolites that engage TLRs or utilizing TLR agonists/antagonists may recalibrate immune responses and restore bone marrow microenvironment homeostasis.

Furthermore, microbial metabolites such as SCFAs, bile acids, and tryptophan derivatives serve as key mediators linking gut microbiota to hematopoiesis and immune regulation. Alterations in these metabolites have been implicated in hematopoietic injury models, such as benzene-induced toxicity, where dysbiosis leads to reduced butyrate and indole-3-propionic acid levels, correlating with hematopoietic impairment (11). Therapeutic restoration of such metabolites, either through dietary supplementation, probiotic administration, or metabolic pathway modulation, could mitigate hematopoietic damage and inflammation. Additionally, microbiota-derived products like phosphatidylcholine and γ-glutamylalanine have been identified to stimulate type I interferon signaling, which supports normal hematopoiesis and may counteract antibiotic-induced bone marrow suppression (18).

In summary, the therapeutic prospects of targeting the gut microbiota to regulate hematopoiesis encompass multifaceted approaches: direct modulation of microbial communities via probiotics, prebiotics, and fecal transplantation; harnessing microbial metabolites to influence hematopoietic niche and immune signaling; and regulating TLR-mediated inflammatory pathways to maintain bone and marrow homeostasis. These strategies hold promise for treating hematopoietic disorders, ameliorating chemotherapy-induced immunosuppression, and managing age-related hematopoietic decline. Future clinical translation will require rigorous characterization of microbiota-host interactions, identification of key microbial taxa and metabolites, and development of precise interventions tailored to individual microbiome profiles (11, 18, 35–37, 45, 50, 100, 101).

#### 2.4.3 Future research directions and technological applications

Future research into the gut-bone marrow axis stands to benefit profoundly from the application of advanced single-cell omics and multi-omics integration technologies. scRNA-seq, single-cell epigenomics, and spatial transcriptomics can dissect the cellular heterogeneity and dynamic states of HSPCs, immune cells, and stromal components within the bone marrow microenvironment, revealing how gut-derived signals modulate their functions at unprecedented resolution. For example, studies have demonstrated that gut microbiota-derived metabolites such as SCFAs influence regulatory T cell (Treg) expansion and osteoblast differentiation, yet the precise molecular pathways and cell-specific responses remain incompletely characterized (39, 61). Single-cell approaches can unravel these complex interactions by profiling transcriptional and epigenetic changes in bone marrow cells upon exposure to microbial metabolites or dysbiosis. Moreover, integrating multi-omics data—including metagenomics, metabolomics, proteomics, and immunophenotyping—can provide a holistic view of the gut-bone marrow crosstalk. For instance, metabolomic profiling combined with 16S rRNA sequencing has elucidated how alterations in gut microbiota composition correlate with changes in circulating metabolites that regulate bone marrow hematopoiesis and immune cell differentiation (102, 103). Such integrative analyses can identify key microbial taxa, metabolites, and host signaling pathways that orchestrate hematopoietic remodeling and immune modulation. Furthermore, spatial multi-omics can map the localization of microbial metabolites and immune cells within the bone marrow niche, providing insights into microenvironmental regulation. These technologies also enable the study of pathological conditions, such as osteoporosis, leukemia, and inflammatory diseases, where gut microbiota perturbations contribute to disease progression via the gut-bone marrow axis (28, 104). Ultimately, leveraging single-cell and multi-omics integration will facilitate the identification of novel biomarkers and therapeutic targets, advancing precision medicine approaches that modulate the gut microbiome or its metabolites to restore bone marrow homeostasis and immune function.

An emerging frontier in gut-bone marrow axis research involves elucidating the synergistic regulation of hematopoiesis by the gut microbiota in concert with the neuroendocrine system. The neuro-immune-endocrine axis within bone marrow microenvironments has been recognized as a critical modulator of bone remodeling and immune cell dynamics, with sympathetic and parasympathetic signaling influencing osteoclastogenesis and macrophage polarization (61). Gut microbiota-derived metabolites such as SCFAs and polyamines have been shown to impact bone immunity through epigenetic and receptor-mediated pathways, suggesting a complex interplay between microbial signals and neuroendocrine factors. However, the precise mechanisms by which gut microbes and neuroendocrine signals coordinate to regulate HSC fate and immune cell trafficking remain largely unexplored. Recent evidence indicates that gut microbiota can modulate systemic hormone levels, including serotonin and leptin, which in turn affect bone homeostasis via central nervous system pathways and bone marrow adiposity regulation (39, 61). Additionally, the gut-brain-bone axis concept posits that vagus nerve-mediated gut signaling enhances osteogenic pathways, integrating neural inputs with microbial and immune cues. Investigating how microbial metabolites influence neuroendocrine receptors on bone marrow stromal and hematopoietic cells could uncover novel regulatory circuits. For example, studies have linked microbial modulation of bile acid metabolism to systemic hormonal changes that impact hematopoiesis and inflammation (97, 105). Furthermore, the role of microbial extracellular vesicles in transmitting signals to neural and bone marrow compartments represents a promising area for mechanistic studies (106). Understanding these neuro-microbial interactions may reveal how stress, diet, and microbial dysbiosis converge to disrupt hematopoietic homeostasis and contribute to diseases such as osteoporosis, leukemia, and inflammatory disorders. Future research integrating neurobiology, microbiology, and hematology, potentially employing advanced imaging, neuronal tracing, and functional genomics, will be essential to decode this complex regulatory network. Such insights could pave the way for innovative therapies targeting the gut microbiota-neuroendocrine axis to modulate bone marrow function and systemic immunity (**Figure 4**).

**Figure 4:**
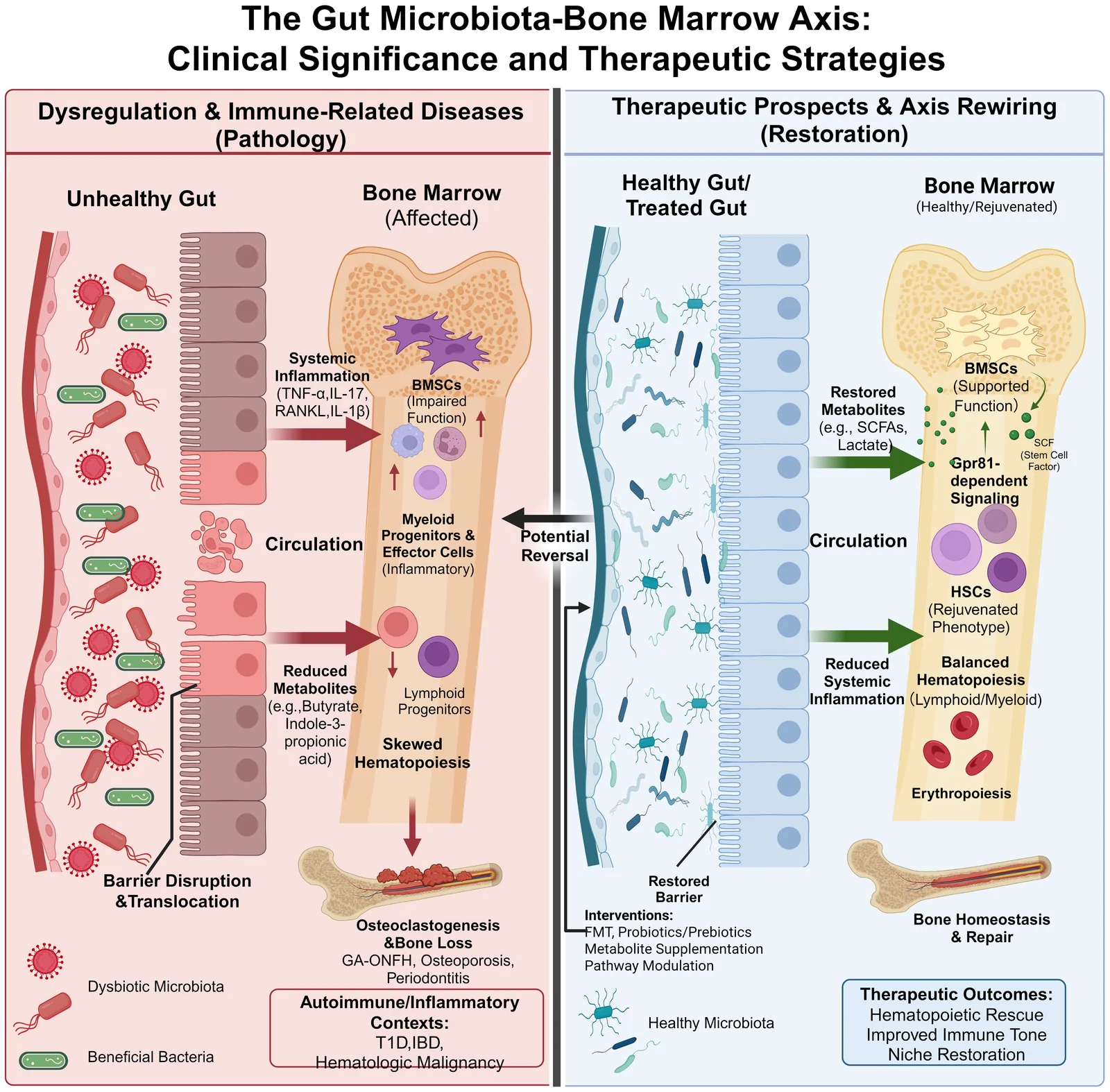
Clinical implications and therapeutic targeting of the gut microbiota–bone marrow axis. Dysregulation of the gut microbiota–bone marrow axis promotes systemic inflammation, myeloid-biased hematopoiesis, osteoclast activation, and immune imbalance, contributing to immune-related and bone metabolic diseases. Therapeutic strategies, including probiotics, prebiotics, fecal microbiota transplantation, microbial metabolite supplementation, and modulation of Toll-like receptor signaling, restore microbial homeostasis and recalibrate hematopoietic and immune function. Integration of single-cell and multi-omics approaches enables precise identification of microbial, metabolic, and immune targets, supporting the development of personalized interventions to restore bone marrow homeostasis and systemic immune balance. Created in BioRender. (2026) https://BioRender.com/z8u6kyz.

## 3 Conclusion

In conclusion, the intricate interplay between the gut microbiota and bone marrow hematopoiesis represents a rapidly evolving frontier in immunology and bone biology. From an expert perspective, this review underscores how the gut microbiome exerts profound influence on bone marrow HSC function and immune cell lineage commitment through both direct metabolic products and indirect modulation of the bone marrow microenvironment. This dual mechanism highlights the complexity of the gut-bone marrow axis, where microbial metabolites act as signaling molecules that shape hematopoietic niches and immune cell differentiation pathways.

Central to this crosstalk is the TLR signaling pathway, which functions as a pivotal molecular bridge linking gut microbial signals to systemic immune responses and bone metabolism. The TLR-mediated regulation of systemic inflammation and myeloid-biased hematopoiesis illustrates how microbial recognition pathways integrate environmental cues to influence bone marrow output. This insight reconciles diverse research findings by positioning TLR signaling as a key node where microbial, immune, and skeletal systems converge, thus providing a unified framework to understand their interdependence.

Moreover, the developmental trajectory of gut-associated B cell populations during early life stages and their role in establishing immune memory offers critical perspectives on immune homeostasis. Understanding how early microbial colonization shapes B cell ontogeny and memory formation not only deepens our grasp of immune system maturation but also suggests windows of opportunity for therapeutic intervention to promote long-term immune stability.

Importantly, dysregulation of the gut-bone marrow axis has been increasingly implicated in a spectrum of immune and bone metabolic disorders, including chronic inflammatory diseases and osteoporosis. This pathogenic link emphasizes the translational potential of targeting gut microbiota and associated signaling pathways. Therapeutic strategies aimed at modulating microbial composition or blocking maladaptive TLR signaling hold promise for restoring hematopoietic balance and mitigating disease progression.

Looking forward, advancing this field necessitates integrative approaches that combine multi-omics technologies—such as metagenomics, metabolomics, and single-cell transcriptomics—with rigorous functional validation. Such comprehensive analyses will be essential to unravel the precise molecular mechanisms by which gut microbiota regulate bone marrow hematopoiesis and immune modulation. This knowledge will be instrumental in driving the development of precision medicine strategies tailored to individual microbial and immunological profiles.

In summary, the gut microbiota-bone marrow axis emerges as a critical regulator of hematopoietic and immune homeostasis, offering novel insights and therapeutic avenues. Balancing the diverse research perspectives—from microbial ecology to molecular immunology and clinical pathology—enables a holistic understanding of this complex system. Continued interdisciplinary research will be key to translating these findings into effective interventions that harness the gut microbiome’s potential to optimize bone marrow function and immune health.
